# Immunotherapy for Cutaneous Squamous Cell Carcinoma in Aging Societies: Integrating Immunosenescence and Geriatric Oncology Perspectives

**DOI:** 10.3390/cancers18050749

**Published:** 2026-02-26

**Authors:** Shigeto Matsushita, Kazuyasu Fujii, Megumi Aoki

**Affiliations:** 1 Department of Dermato-Oncology, NHO Kagoshima Medical Center, Kagoshima 892-0853, Japan; kazuyasu.fujii@med.kindai.ac.jp (K.F.); aokm75@yahoo.co.jp (M.A.); 2 Department of Dermatology, Kindai University Hospital, Osaka 590-0197, Japan

**Keywords:** cutaneous squamous cell carcinoma, immune checkpoint inhibitors, programmed cell death protein 1 inhibitors, systemic therapy, geriatric oncology principles

## Abstract

Background/Objectives: Cutaneous squamous cell carcinoma (cSCC) is the second most common skin cancer worldwide, with a steadily increasing incidence in aging populations, particularly among older and immunocompromised individuals. Early-stage cSCC disease is usually managed with surgery or radiotherapy; however, advanced or unresectable cases remain therapeutically challenging. The advent of immune checkpoint inhibitors (ICIs), especially programmed cell death protein 1 inhibitors, such as cemiplimab and pembrolizumab, has markedly improved outcomes in advanced cSCC. Nevertheless, resistance to immunotherapy, management of frail or super-aged patients, and optimal integration of systemic therapy with radiotherapy continue to pose important clinical questions. Methods: This review provides an updated overview of the current treatment strategies for cSCC, with particular attention to recent advances in systemic therapy and the application of geriatric oncology principles. We emphasize the considerations relevant to East Asian practices, especially in Japan, where the demographic shift toward a super-aged society magnifies the need for tailored approaches. Results: Although most pivotal clinical trials have been conducted in Western populations, emerging real-world data from East Asia have demonstrated comparable efficacy and safety of ICIs, underscoring the global relevance of immunotherapy in diverse patient groups. By incorporating a structured frailty assessment, individualized treatment goals, and proactive management of immune-related adverse events, this review highlights practical strategies for optimizing cSCC care in older patients. Conclusions: Future directions include predictive biomarker development; refinement of treatment sequencing; and multidisciplinary collaboration among oncology, dermatology, and geriatrics to adapt cSCC management to aging societies.

## Simple Summary

Cutaneous squamous cell carcinoma is one of the most common skin cancers, especially in older adults. With the rapid aging of societies, particularly in East Asia, the number of very old patients with this disease is increasing. Although surgery and radiotherapy can cure early-stage tumors, advanced or unresectable cases have been difficult to manage. In recent years, new medicines called immune checkpoint inhibitors have offered major improvements, helping many patients live longer with fewer side effects than those of older treatments. This review explains how these drugs are being used in patients with advanced disease, especially in older groups who may have other health problems or reduced physical strength. We also highlight the importance of careful evaluation of frailty, daily function, and other age-related issues when deciding on treatment. We aim to guide doctors and researchers in adapting skin cancer care to the realities of aging societies.

## 1. Introduction

Cutaneous squamous cell carcinoma (cSCC) is the second most common skin cancer worldwide. Its incidence has been increasing in parallel with population aging, particularly among individuals aged ≥65 years [1,2]. Striking geographic and ethnic disparities exist: the incidence is 270 per 100,000 person-years in Australia [3] but remains significantly lower in East Asia (e.g., approximately 3–4 per 100,000 in Japan) [4]. However, rapidly aging societies in East Asia are now experiencing a disproportionate increase in disease burden; for instance, the incidence of cSCC nearly doubled between 1999 and 2019 [5].

In Japan, nationwide registry data from the National Cancer Registry (NCR)—which has systematically captured cancer incidence since 2016—have confirmed that cSCC accounts for approximately 44% of all skin cancers, with a particularly sharp increase among the older population [4]. Because not all keratinocyte carcinomas are fully ascertained in the NCR, the actual burden of cSCC is likely underestimated. Furthermore, clinical data have shown that patients aged ≥90 years represent a substantial and growing subgroup; in a multicenter surgical series, one-third of all cSCC cases occurred in nonagenarians, who exhibited a significantly higher recurrence risk despite standard treatment [6]. Although cSCC generally has a lower case-fatality rate than melanoma, its burden in older adults is substantial; for example, the disability-adjusted life-year rate in the 70–74-year age group is approximately 74.59 per 100,000 population, underscoring its significant impact on aging societies [7]. These epidemiological observations highlight the urgent need to adapt cSCC management strategies to the realities of super-aged societies, particularly in East Asia, while recognizing the regional and ethnic disparities in incidence and outcomes. Therefore, a deeper understanding of the biological mechanisms underlying cSCC development is essential. In particular, age-associated changes in the immune system, commonly referred to as immunosenescence, play a critical role in the pathogenesis of cSCC and the response to emerging therapies, such as programmed cell death protein 1 (PD-1) inhibitors. Accordingly, this review focuses on the age- and region-specific challenges of cSCC management in older patients in East Asia, with a particular emphasis on frailty, comorbid conditions, and the safe use of immunotherapy in Japan’s super-aged society, where local data remain limited.

This review was conducted as a narrative literature synthesis. We searched PubMed and screened major dermatology/oncology guidelines (Japanese Dermatological Association, the Journal of the American Academy of Dermatology, the European Association of Dermato-Oncology, the National Comprehensive Cancer Network) for publications related to “cutaneous squamous cell carcinoma,” “PD-1 inhibitors,” “older adults,” “geriatric oncology,” and “East Asia/Japan” from January 2010 to October 2025, with particular emphasis on studies published after 2015 corresponding to the era of immune checkpoint inhibitors (ICIs). No formal systematic review methods were applied. Reference lists of relevant studies were manually screened to identify additional sources. Given the narrative design, formal inclusion/exclusion criteria, protocol registration, and quantitative risk-of-bias assessment were not performed; therefore, the synthesis reflects a structured qualitative interpretation of the available evidence rather than a meta-analytic evaluation.

## 2. Biology and Risk Factors Relevant to Cutaneous Squamous Cell Carcinoma (cSCC) in the Older Population

cSCC predominantly affects older adults, and the aging immune system (“immunosenescence”) can influence tumor behavior and treatment outcomes. Immunosenescence is the progressive decline in immune function with age and is characterized by systemic changes that foster tumor immune evasion [8]. This section explores key age-related immunological changes, including T-cell dysfunction, altered antigen presentation, accumulation of suppressive cells, and inflammatory shifts, and how they affect the efficacy and safety of PD-1 inhibitor immunotherapy in cSCC. We draw on evidence from cSCC and related cancers to highlight the tumor microenvironment (TME) in older patients and the clinical outcomes of immune checkpoint blockade in this population.

### 2.1. Immunosenescence: Age-Related Immune Changes

Aging is associated with broad immune remodeling, which can impair antitumor immunity. Thymic involution and bone marrow changes lead to reduced naïve T-cell output, skewing toward memory and senescent T-cell populations [9,10,11]. Thus, older individuals have fewer fresh T cells that respond to new antigens and more terminally differentiated or exhausted T cells. Aged T cells often upregulate inhibitory receptors, such as PD-1, T-cell immunoglobulin and mucin-domain containing-3, and lymphocyte activation gene-3 [12]. These age-related immune alterations may affect the responsiveness and toxicity profiles of ICIs in older adults [13]. Functionally, older T cells exhibit a diminished proliferative capacity and impaired cytotoxicity [14]. In parallel, age-associated defects in antigen-presenting cells (APCs), including dendritic cells, result in reduced costimulatory activity, which may compromise the efficient priming of T-cell responses [14]. The cytotoxic function of natural killer cells also declines with age [14], further limiting the ability of the innate immune system to control tumors.

Immunosenescence is also marked by myeloid skewing of hematopoiesis, leading to the increased production of myeloid cells at the expense of lymphoid cells [15]. This can translate into higher levels of immunosuppressive myeloid-derived suppressor cells (MDSCs) and M2-polarized macrophages in older individuals, which actively dampen T-cell responses in the TME [16]. Additionally, aging tissues accumulate senescent cells (e.g., fibroblasts) that secrete the senescence-associated secretory phenotype (SASP), including interleukin [IL]-6, IL-1 family cytokines, and transforming growth factor [TGF]-β, promoting chronic low-grade inflammation (“inflammaging”) [16,17]. For example, regulatory T cells (Tregs; CD4^+^FOXP3^+^), which restrain antitumor immunity, increase in frequency with age in mice and in certain human tissues [18]. Conversely, the pool of antigen-specific effector T cells may be present in normal numbers in an aged host but fail to migrate into peripheral tissue efficiently. Notably, older individuals show impaired homing of circulating T cells to the skin [19], undermining local tumor surveillance. Taken together, these changes—T-cell exhaustion, waning APC function, Treg/MDSC accumulation, and cytokine imbalances—create an immune landscape in older patients that can be tipped toward tumor tolerance and the evasion of immune surveillance.

In cSCC, age-related immune remodeling may impair immune surveillance, whereas the typically high ultraviolet-driven tumor mutational burden (TMB) may sustain responsiveness to PD-1 blockade; nevertheless, clinical outcomes remain strongly influenced by functional status and frailty.

### 2.2. Tumor Microenvironment in the Older Population

The TME in older patients with cSCC reflects intrinsic tumor factors and an aged host milieu. Chronic ultraviolet (UV) exposure, which is common in the older population, not only drives the high mutational burden of cSCC but also causes cumulative dermal damage and senescence in the surrounding stroma [20]. Senescent fibroblasts in aged skin acquire a pro-inflammatory, pro-tumor phenotype: they secrete growth factors (e.g., IL-6, IL-8, vascular endothelial growth factor) and matrix-degrading enzymes that promote cancer cell proliferation, angiogenesis, and invasion. Tumor cells can “educate” resident fibroblasts into cancer-associated fibroblasts resembling senescent cells, suggesting that an aged stromal environment may be especially permissive for tumor progression [20]. Moreover, TGF-β, often elevated in photoaged skin [21], can suppress effective antitumor immunity [16] and has been implicated in resistance to checkpoint inhibitors by excluding T-cell infiltration [22]. In older patients with cSCC and related squamous cancers, the TME is often enriched with immunosuppressive elements, such as Tregs. Azzimonti et al. reported that Treg infiltration was greater in cSCC than in normal skin, with particularly high levels observed in moderately to poorly differentiated (more invasive) lesions. They also showed that aggressive SCC subtypes exhibited a reduced CD8+/Treg ratio, underscoring the association between Treg predominance and tumor aggressiveness [23]. An aged TME may also show reduced T-cell trafficking; effector T lymphocytes in older individuals infiltrate the skin less efficiently, partly because of diminished endothelial adhesion molecule expression and altered chemokine signaling [19], a phenomenon likely relevant to cSCC.

Despite these challenges, some aspects of the aging TME may paradoxically favor responses to immunotherapy. Chronic antigenic stimulation in the older population can lead to clonal expansion of certain T cells, including tumor-specific clones [18], and cSCC tumors in older patients often harbor extremely high neoantigen loads because of UV-induced DNA damage [24]. A high TMB is associated with improved survival, and in some studies, greater responsiveness to PD-1 blockade [25] suggests that the abundance of neoantigens may, at least in part, counterbalance age-related immune senescence. Additionally, although systemic Tregs increase with age, intratumoral Treg density is not necessarily higher in older patients. In melanoma, FOXP3^+^ Tregs were found to be less abundant in tumors from older individuals than in those from younger individuals, resulting in a more favorable effector (CD8^+^) to Treg ratio within the TME [26]. This may reduce local immunosuppression and improve the efficacy of checkpoint inhibitors in older tumors. Overall, the aged cSCC microenvironment is a combination of heightened tumor antigenicity and immunosuppressive host factors. Understanding this balance is the key to predicting how PD-1 inhibitor therapy should be administered to geriatric patients.

### 2.3. Risk Factors Beyond UV Exposure in Older Adults

Although chronic UV radiation is the dominant driver of cSCC development, several additional risk factors—particularly relevant in aging populations—deserve consideration. Genetic predisposition syndromes, such as xeroderma pigmentosum and oculocutaneous albinism, markedly impair DNA repair, thereby increasing the lifetime risk of cSCC. In addition, human papillomavirus-associated cSCC, especially in periungual and anogenital sites, may play an underrecognized role in older adults with long-standing immunosuppression. Medication-related photosensitization is another growing contributor: long-term use of hydrochlorothiazide has been reportedly associated with an increased risk of keratinocyte carcinoma, including cSCC, and a dose–response relationship has been suggested in observational studies. Evidence for other thiazide-class or antihypertensive agents appears less consistent. However, these findings derive primarily from observational data and should be interpreted with appropriate caution. These non–UV-related factors are particularly relevant in super-aged societies, where polypharmacy, extended life expectancy, and multimorbidity may cumulatively elevate cSCC risk beyond UV exposure alone.

**Figure 1:**
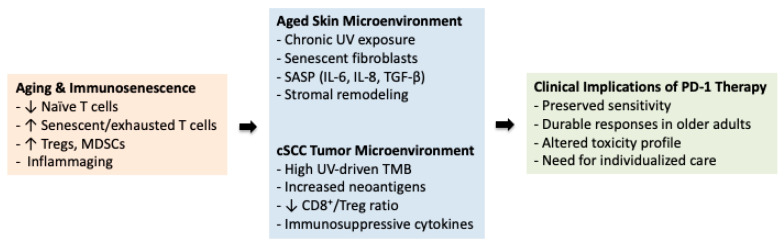
Immunosenescence and the tumor–immune interface in cutaneous squamous cell carcinoma (cSCC). Aging is associated with immunosenescence, characterized by reduced naïve T-cell output, accumulation of senescent or exhausted T cells, expansion of regulatory T cells and myeloid-derived suppressor cells, and chronic low-grade inflammation (“inflammaging”). In chronically sun-exposed skin, senescent fibroblasts and stromal remodeling promote a tumor-permissive microenvironment through the senescence-associated secretory phenotype, including interleukin (IL)-6, IL-8, and transforming growth factor-β. Cutaneous squamous cell carcinoma in older adults typically exhibits a high ultraviolet-driven tumor mutational burden (TMB) and altered immune cell balance. Despite age-related immune remodeling, high tumor immunogenicity allows preserved sensitivity to PD-1 blockade in many older patients. Arrows indicate the conceptual progression from age-related immune changes to tumor microenvironment alterations and their clinical implications.

To synthesize the key mechanisms through which age-related immune remodeling and tumor–stromal interactions influence cSCC biology and immunotherapy outcomes in older adults, the major concepts are schematically summarized in Figure 1.

## 3. Standard Local Therapies and Their Limits

cSCC is typically managed with local therapies, such as surgical excision, which is often curative for most primary tumors, and radiotherapy as an adjunct or alternative when surgery is not feasible. However, these local treatments have important limitations in a subset of patients. Although >90% of patients with cSCC exhibit relatively indolent behavior, a minority progress to advanced disease, locally extensive or metastatic, that cannot be adequately controlled by surgery or radiotherapy [27]. High-risk tumors, such as large, deeply invasive lesions or those with perineural invasion, are associated with an increased risk of recurrence and metastasis [28,29]. Overall, approximately 5% of cSCC develop metastases [28,29]. In advanced cSCC, curative resection may be impossible or may be associated with unacceptable morbidity [30]. Moreover, not all patients can tolerate aggressive surgery, particularly older individuals or those with significant comorbidities [31,32]. Although radiotherapy can improve local control or serve as the primary treatment for inoperable cases, it is generally insufficient when the disease is widespread or metastatic. These limitations are particularly pronounced in older patients for whom comorbidities, frailty, or reduced tolerance to surgery and radiotherapy often further restrict curative options. In such situations, local interventions alone are inadequate, and systemic therapy is necessary for disease control [30,33].

Taken together, these findings indicate that chronological age or comorbidity does not consistently diminish ICI efficacy in cSCC; rather, poor PS (Eastern Cooperative Oncology Group [ECOG] PS ≥ 2) is the most consistent predictor of inferior survival.

## 4. Systemic Treatment Strategies for Advanced cSCC

Until recently, systemic options for advanced cSCC were limited and generally ineffective. Cisplatin-based chemotherapy, often combined with 5-fluorouracil or taxanes, and the epidermal growth factor receptor (EGFR) inhibitor cetuximab have historically been used off-label in cSCC, drawing on regimens for head and neck squamous cell carcinoma [34]. These approaches achieved only modest responses, with an overall response rate of approximately 20–30% and a median response duration of approximately 5 months, and were further constrained by short-lived responses and significant toxicity [35]. Nevertheless, EGFR inhibition remains an option for patients who are ineligible for immunotherapy, such as transplant recipients or those with uncontrolled autoimmune diseases. In addition, ongoing translational research is exploring molecularly targeted approaches based on frequent alterations in pathways, such as NOTCH, MAPK, and PI3K in cSCC [36,37], although these remain investigational. PD-1 inhibitors have become the standard first-line therapy; however, targeted therapies retain a niche role and may expand as precision oncology advances.

Therefore, the current European guidelines recommend that treatment decisions for advanced cSCC be made in a multidisciplinary setting, explicitly weighing toxicity risks along with patient age, frailty, and comorbidities [38]. More recently, ICIs, especially PD-1 inhibitors, have fundamentally changed this landscape. Given the high UV-induced mutational burden of cSCC and its association with impaired immune surveillance, PD-1 blockade is a biologically rational and clinically effective strategy [39]. ICIs have markedly improved outcomes, with more favorable tolerability than that of prior systemic therapies.

### 4.1. Evidence from Clinical Trials

PD-1 inhibitors are currently established as standard systemic therapies for advanced or unresectable cSCC. In pivotal trials, cemiplimab achieved an objective response rate (ORR) of approximately 46% with durable responses. Updated analyses demonstrated a 24-month overall survival rate of 73% in advanced cSCC (both locally advanced and metastatic disease) [33,40,41]. Pembrolizumab showed comparable efficacy, with ORRs of 35.2% (recurrent/metastatic) to 50% (locally advanced) and a median duration of response (DOR) that was not reached [42,43]. In the CARSKIN trial, pembrolizumab achieved an ORR of 42% in first-line unresectable disease [42]. Exploratory analyses suggested numerically higher response rates in programmed death-ligand 1 (PD-L1)-positive tumors; however, meaningful responses were also observed in PD-L1–negative cSCC, indicating that PD-L1 expression should be considered a non-determinative biomarker. Pacmilimab, tested in a small cSCC cohort (*n* = 14), showed an ORR of 36%, including one complete response; however, median DOR and survival outcomes were not formally reported, although several responses remained ongoing beyond 12 months [44]. Both cemiplimab and pembrolizumab demonstrated manageable safety, with grade ≥ 3 treatment-related adverse events in 7–17.1% of patients. Table 1 summarizes the key trials.

Predictive biomarkers for ICIs in cSCC remain under active investigation, and no biomarker has yet been formally validated for treatment selection in routine clinical practice. PD-L1 expression has been explored as a potential biomarker in clinical trials; however, clinically meaningful responses have also been observed in PD-L1–negative tumors, and current evidence does not support its use as a standalone biomarker for treatment selection [41,43]. Similarly, high TMB, commonly observed in ultraviolet-driven cSCC, provides a biologically plausible rationale for ICI responsiveness, but standardized cut-offs and prospective validation—particularly in very elderly populations—are lacking. Emerging exploratory markers, including immune gene-expression signatures, CD8^+^ T-cell density, regulatory T-cell infiltration, and circulating cytokines associated with immunosenescence (e.g., IL-6 and other components of the senescence-associated secretory phenotype), remain investigational. At present, treatment decisions in advanced cSCC are guided primarily by clinical factors—tumor stage, resectability, performance status, and comorbidities—rather than biomarker stratification.

A structured multidisciplinary approach is essential for patients with resectable but high-risk cSCC, such as those with large tumors, deep invasion, perineural involvement, or high-risk anatomic locations. Management typically begins with a review by the multidisciplinary tumor board, incorporating dermatology, surgical oncology, radiation oncology, and, when appropriate, geriatric assessment. For patients with adequate performance status, neoadjuvant PD-1 inhibitor therapy may be considered in selected cases based on early-phase clinical studies suggesting high pathological response rates; however, this strategy remains unconfirmed in randomized trials (see Section 7 for a detailed discussion). Therefore, neoadjuvant PD-1 therapy should currently be regarded as investigational and applied within multidisciplinary discussion rather than as an established standard of care. After surgical excision with histologic margin assessment, postoperative (adjuvant) treatment should be individualized based on pathological features: surgery alone for low-risk margins, adjuvant radiotherapy for perineural invasion or close/positive margins, and consideration of adjuvant PD-1 blockade in selected high-risk cases, informed by emerging randomized evidence in the adjuvant setting suggesting potential disease-free survival benefit. This algorithm represents a pragmatic synthesis of available evidence and expert consensus rather than a guideline-mandated standard.

### 4.2. Insights from Real-World Evidence

Real-world cohorts provide complementary insights into the feasibility of using PD-1 inhibitors in broader patient populations. Across Europe, real-world cemiplimab cohorts have reported ORRs ranging from 32% to 76.7%, with 1-year survival rates generally around 60% in larger series [45,46,47,48]. In a German multicenter study, 1-year PFS was 58.8%, and treatment discontinuation due to toxicity occurred in <10% of patients [45]. In a French multicenter cohort (*n* = 245), the 1-year OS rate was 63.1%, with grade ≥ 3 immune-related adverse events in 9% of patients [46]. Smaller cohorts of older or frail patients showed greater heterogeneity, including higher discontinuation rates in selected elderly populations [47,48]. The US series also demonstrated activity, with ORRs 26.7–42.3% and responses linked to TMB and head/neck primaries [49,50]. In Latin America, nivolumab produced an ORR of 58.3%, with no complete response; however, its feasibility has been demonstrated [51]. In Australia, a large multicenter cohort (*n* = 286; median age, 75.2 years) showed an ORR of 60% and a 1-year OS rate of 78%, including 31% immunocompromised patients; ECOG performance status (PS) ≥ 2 was observed in 21% of the patients [52]. A separate single-center study (*n* = 53; median age, 81.8 years) reported an ORR of 57% (complete response, 33%) with a 1-year OS rate of 63% and confirmed worse outcomes in patients with ECOG PS ≥ 2 [53]. Japanese data, although limited (*n* = 14), showed an ORR of 57.1% and a 1-year OS rate of 73.1% [54]. However, because of the limited sample size of this cohort, these early findings should be interpreted with caution. Table 2 presents a summary of regional real-world outcomes.

Taken together, these findings indicate that chronological age or comorbidity does not consistently diminish ICI efficacy in cSCC; rather, poor PS (ECOG PS ≥ 2) is the most consistent predictor of inferior survival.

## 5. Geriatric Oncology Principles in Practice

Older patients with cSCC often present with unique challenges that necessitate a geriatric oncology approach. Chronological age alone is a poor surrogate of a patient’s true physiological reserve [55,56]. Many patients in their 70s or 80s are robust and can tolerate therapy well, whereas others are frail, have a multidimensional state of decreased functional reserve, and are at a higher risk of adverse outcomes. Standard PS scores (ECOG PS or Karnofsky) often miss these nuances, as comprehensive geriatric assessments reveal hidden vulnerabilities even in “fit” older adults [55,56]. For example, 69% of older patients with cancer with normal PS had at least one deficit, commonly polypharmacy (≥9 drugs in 43%) or multiple comorbidities (≥4 in 25%) [57]. These findings underscore that routine oncology evaluations may underestimate patient risk, and guidelines recommend geriatric screening for those aged ≥65 years starting systemic therapy. Assessing domains, such as function, comorbidities, cognition, nutrition, and social support, provides individualized care [55,56].

A critical implication of these hidden frailties is their impact on immunotherapy tolerance and safety. ICIs, such as anti-PD-1 antibodies, have become key systemic treatments for advanced cSCC, especially in older patients. Chronological age alone does not increase immune-related adverse events (irAEs) [58,59,60]; instead, frailty and comorbidity drive outcomes. For instance, mild toxicities, such as diarrhea, may precipitate dehydration or kidney injury in frail octogenarians, and steroids for irAEs may trigger delirium or infection [61,62]. In real-world analyses, irAE incidence was comparable to trial data, but patients with ECOG PS ≥ 2 had worse survival [53]. Similarly, frail patients with melanoma had higher hospitalization rates despite similar rates of grade ≥ 3 irAEs [63].

Polypharmacy further amplifies risks, as >40% of older adults take ≥9 drugs daily, increasing the likelihood of adverse drug events and drug–drug interactions that require careful consideration when initiating immunotherapy [57,61]. Multidisciplinary coordination among geriatricians, pharmacists, and caregivers is crucial because even low-grade toxicities may require early intervention in patients with frailty.

Importantly, chronological age alone should not automatically preclude immunotherapy; treatment decisions must be individualized, particularly in very elderly or frail patients. Many older adults (median age, 70s) were enrolled in pivotal PD-1 trials, which revealed ORRs of approximately 40–50% [33,42,43]. Real-world cohorts have reported broadly comparable response rates, with ORRs of approximately 50% in a French expanded-access program [46], approximately 60% in an Australian series [52], and 76.7% in a frail Italian cohort [48]; however, these findings derive from heterogeneous observational studies with varying performance status and comorbidity profiles. Responses were observed even in immunosuppressed patients, suggesting that clinically meaningful responses can occur, although data remain limited and heterogeneous. The strongest predictor of survival is functional status; in France, the 1-year OS rate was 73% with ECOG PS 0–1 versus 36% with ECOG PS ≥ 2 [46]. Similarly, US data showed that ECOG PS ≥ 3, not age ≥ 75 years, predicted worse survival [64]. Thus, a fit 80-year-old may benefit as much as younger patients, whereas a frail counterpart may face a higher risk. The challenge is to identify the modifiable vulnerabilities and intervene accordingly.

Integrating geriatric principles, screening for vulnerabilities with tools such as the Geriatric-8 (G8), optimizing comorbidities and medications, early irAE management, and aligning goals of care maximizes benefits while minimizing harm [65,66,67]. Shared decision-making is essential for tailoring therapy to patient priorities and balancing cure with palliation.

**Figure 2:**
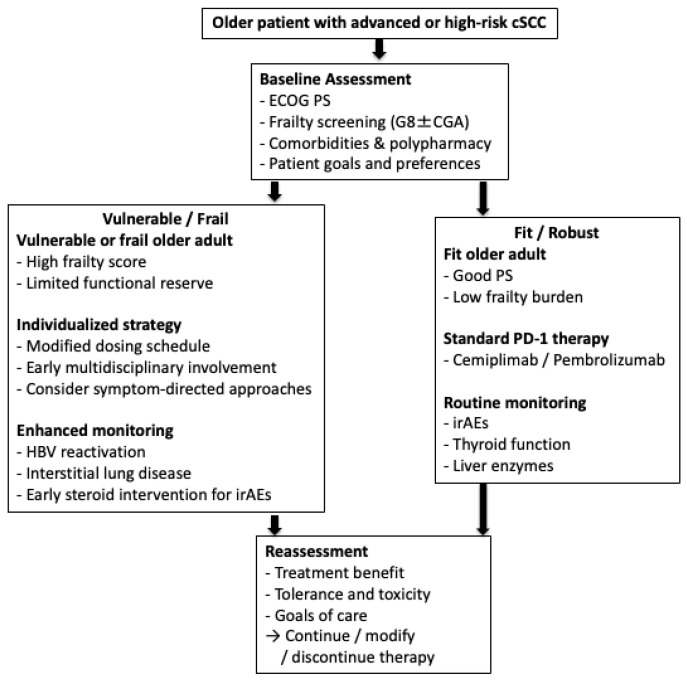
Geriatric decision pathway for PD-1 inhibitor therapy in older adults with cutaneous squamous cell carcinoma (cSCC). Management of advanced or high-risk cSCC in older adults begins with a comprehensive baseline assessment that includes performance status, frailty screening (e.g., G8 and/or comprehensive geriatric assessment), comorbidities, polypharmacy, and patient care goals. Fit or robust older adults may proceed with standard PD-1 inhibitor therapy with routine monitoring for immune-related adverse events. In contrast, vulnerable or frail patients may benefit from individualized treatment strategies, early multidisciplinary involvement, and enhanced safety monitoring. Chronological age alone should not preclude immunotherapy; functional status and frailty should guide treatment decisions. Arrows indicate the clinical decision-making pathway based on baseline assessment and functional status.

By applying these practices, clinicians can safely extend the advances in immunotherapy to a growing population of older patients with cSCC. Selected octogenarians and nonagenarians may achieve favorable outcomes, although available evidence is primarily derived from observational cohorts [68]. This emphasis must remain on physiological rather than on chronological age [69]. Tailoring care in this manner will ensure that advances in immunotherapy translate into real-world survival and quality-of-life gains for this vulnerable group. A practical geriatric decision pathway integrating frailty assessment, treatment selection, and safety monitoring for PD-1 inhibitor therapy in older adults with cSCC is shown in Figure 2.

## 6. Regional Perspectives: East Asia and Japan

As summarized in Section 4.2, most real-world evidence to date has come from Europe, the United States, and Australia, where outcomes with PD-1 inhibitors closely mirror those observed in clinical trials. However, data from East Asia are limited. In Japan, early real-world experiences have recently been reported, demonstrating response rates and safety outcomes that are consistent with international trial data [54]. These findings indicate that Japanese patients with cSCC respond to immunotherapy similarly to their Western counterparts, with no unexpected safety concerns.

### 6.1. Optimizing Treatment Sequences

The advent of ICIs has shifted treatment paradigms across East Asia. Historically, cytotoxic chemotherapy and EGFR inhibitors, such as cetuximab, were occasionally used in unresectable cSCC, but their antitumor activity was limited, with only modest response rates reported in international studies [34,70]. Given their limited efficacy, these targeted agents have been largely supplanted by ICIs as first-line systemic therapy. Consequently, current practice in Japan and elsewhere favors PD-1 inhibitors as initial therapy for advanced cSCC [54]. Studies have explored how best to sequence or combine these modalities. The recent I-TACKLE study in Italy examined the effects of cetuximab after PD-1 inhibitor failure [71]. This approach achieved a cumulative ORR of 63% using cetuximab to overcome pembrolizumab resistance and elicited responses in 10 of 23 resistant patients (43%). However, this sequential strategy comes at the expense of increased toxicity. EGFR blockade causes skin toxicities; in a phase II cetuximab monotherapy trial, 78% of patients developed an acneiform rash, generally grade 1–2 [34]. Thus, although adding cetuximab may benefit a subset of ICI-refractory patients, clinicians should be mindful of the risk of rash and other EGFR-related adverse effects, which are often low grade and manageable in routine practice.

### 6.2. Tolerability and Unique Considerations in East Asian Populations

Overall, the currently available data suggest that East Asian patients generally tolerate immunotherapy comparably to Western populations, although published regional cohorts are sparse and predominantly retrospective. However, there are ethnic considerations. For example, interstitial lung disease and ICI-related pneumonitis have been reported at numerically higher rates in some Japanese cohorts, which may reflect a higher baseline risk of drug-induced interstitial lung disease in this population [72]. Similarly, immune-related hepatitis, typically presenting as elevations in liver enzyme levels, requires particular vigilance in East Asia, where chronic hepatitis B virus (HBV) infection remains highly prevalent and contributes to the risk background for irAEs; in Japan, attention is paid to HBV reactivation during ICI therapy [73,74].

In response to these risks, oncologists in Japan and neighboring countries carefully screen for latent infections and monitor hepatic function during ICI therapy. Importantly, these factors can be managed with proper precautions and do not preclude effective treatment. In practice, Japanese patients have tolerated ICIs well, with efficacy and safety outcomes broadly consistent with those reported in Western trials, albeit based on small cohorts and no new safety signals observed [54]. This finding is consistent with the broader oncology experience, as studies in Asian lung cancer populations have also demonstrated outcomes similar to those in Caucasian patients [72,75].

Although PD-1 inhibitors are now widely used in advanced cSCC, certain high-risk groups require tailored decision-making. Recipients of solid organ transplants face a substantial risk of allograft rejection with PD-1 blockade, and treatment decisions should involve shared discussions with the transplant team. In such cases, EGFR inhibition (cetuximab), radiotherapy, or local treatments may serve as alternative options when immunotherapy is contraindicated. For older adults with autoimmune diseases, clinicians should anticipate a modestly higher risk of flares and discuss the expected benefits and risks in advance.

In East Asia, proactive safety monitoring is particularly important. HBV screening (HBsAg, anti-HBc, and HBV-DNA when indicated) is recommended before initiating PD-1 inhibitors in accordance with relevant hepatology and oncology society guidelines, particularly in regions with higher HBV prevalence, with antiviral prophylaxis or close monitoring in patients with chronic or resolved infection. Routine assessments should include baseline and every 3–6 weeks, liver and thyroid function tests. Because interstitial lung disease and immune-related pneumonitis have been reported in some Japanese and East Asian cohorts, clinicians should remain vigilant for early respiratory symptoms. A baseline chest CT may be considered in selected high-risk patients to document pre-existing interstitial lung abnormalities and provide a reference for comparison if respiratory symptoms develop during therapy. Early pulmonology consultation, together with timely initiation of corticosteroids, should be undertaken if pneumonitis is suspected. These practical measures support safe delivery of immunotherapy in older and medically complex patients.

Beyond biological and safety considerations, structural aspects of healthcare systems in East Asia also shape immunotherapy implementation. Japan, as a super-aged society with universal health coverage, has a high proportion of patients aged ≥80 years undergoing active cancer treatment. Many older adults present with multimorbidity, polypharmacy, and varying degrees of frailty, which influence therapeutic tolerance more than chronological age alone. In addition, strong coordination between dermatologists, surgeons, radiation oncologists, and community-based primary care physicians facilitates close monitoring and early management of immune-related adverse events. These system-level characteristics may partially explain why real-world outcomes in Japan have been comparable to global benchmarks despite the advanced age of treated populations, although direct comparative analyses are underexplored.

In summary, the regional experience, albeit early, suggests that ICIs are transforming advanced cSCC care in East Asia, just as they have elsewhere. Japan has led regional adoption, reporting response rates on par with global benchmarks. Across East Asia, ICIs are increasingly used for cSCC, and although published data remain limited, there is no evidence of their reduced efficacy. Ongoing research and postmarketing surveillance will refine treatment strategies, but the current findings are encouraging: Japanese and East Asian patients can achieve excellent results with immunotherapy, supporting its role as a standard systemic treatment option in appropriately selected patients across diverse populations. These regional experiences underscore not only the comparable efficacy of immunotherapy in East Asia but also the need for continued refinement of treatment strategies. This naturally leads to broader questions about how cSCC management can evolve in the future, particularly in terms of predictive biomarkers, treatment sequencing, and the integration of geriatric principles into standard care.

## 7. Future Directions and Unmet Needs

One major direction is the development of predictive biomarkers to guide immunotherapy selection, as no robust markers, such as TMB, PD-L1 status, or CD8^+^ T-cell infiltration, have yet been established, despite the high immunogenicity of cSCC [76]. Its tumor mutation burden is among the highest among all cancers, and PD-L1 is frequently expressed [39,76]. Some studies have reported higher response rates in PD-L1–positive tumors; however, responses also occur in PD-L1–negative disease, and prospective validation remains necessary, underscoring the need to validate these biomarkers in prospective studies [42].

Another emerging strategy is neoadjuvant immunotherapy. In a single-arm phase II study of patients with high-risk resectable cSCC, PD-1 blockade achieved approximately 50% pathological complete response and 89% 1-year event-free survival [77]. Although these results are encouraging, randomized comparative data are still lacking.

Recent phase III evidence also supports adjuvant PD-1 blockade after surgery for high-risk cSCC, indicating that perioperative immunotherapy may further improve long-term outcomes [78]. However, long-term overall survival data and evidence in very old or frail subgroups remain limited.

However, several important limitations should be considered when interpreting the current evidence base. First, most pivotal clinical trials and large datasets evaluating PD-1 inhibitors in cSCC were conducted predominantly in Western populations. Hence, they may not fully capture the genetic, environmental, or healthcare characteristics of East Asian patient groups. Second, direct head-to-head randomized comparisons between immunotherapy and conventional modalities are lacking, which restricts definitive conclusions regarding comparative efficacy. Third, robust data for frail, polymorbid, or very old adults—who constitute a large proportion of those with cSCC in real-world settings—is lacking in breadth, and predictive biomarkers capable of guiding patient selection have not been validated. Fourth, real-world evidence is inherently constrained by potential reporting bias, heterogeneous treatment practices, and variable follow-up durations. In addition, functional outcomes, quality of life, and long-term toxicity are insufficiently characterized in older adults, and potentially relevant findings from non-English or regional studies may be underrepresented in narrative syntheses. These limitations underline the need for more inclusive, methodologically rigorous research to refine treatment strategies for cSCC in aging societies. Future studies should prioritize the inclusion of very old and frail populations, standardized outcome reporting, and region-specific analyses to improve the generalizability of findings.

Finally, integrating frailty tools and geriatric assessments into treatment planning is crucial for the predominantly older patients with cSCC [66]; instruments, such as the G8 index, can predict treatment tolerability, including postoperative complications, and Japan-based initiatives advocate incorporating such geriatric assessments in clinical trials to optimize therapy for older patients with cSCC. Multidisciplinary collaboration between geriatric medicine and oncology is essential for optimizing care in super-aged societies, such as Japan.

## 8. Conclusions

In the context of aging societies, particularly in East Asia, where older patients represent a growing majority of cSCC cases, optimizing management requires careful consideration of regional characteristics and age-specific vulnerabilities. ICIs have reshaped the treatment landscape of advanced cSCC, with durable responses observed in selected patients, including carefully evaluated older adults. For optimal implementation, integration of geriatric oncology principles, especially structured frailty assessment and multidisciplinary coordination, should be systematically incorporated into routine decision-making. Although regional real-world data from East Asia are encouraging, there is limited evidence in very old, frail, or highly polymorbid populations. Unresolved questions regarding biomarker-driven selection, perioperative strategies, and long-term outcomes underscore the need for prospective, methodologically rigorous studies to refine treatment strategies in super-aged societies.

## Figures and Tables

**Table 1:** Key clinical trials of PD-1 and PD-L1 inhibitors in advanced cSCC.

Agent/Trial	Phase	Setting	*n*	Median Age (Years)	ORR (%)	CR (%)	Median DOR	Key Survival Outcome	Grade ≥ 3 TRAEs (%)	Reference
Cemiplimab/EMPOWER-CSCC-1	I/II	Locally advanced and metastatic	193 (pooled analysis)	72	46.1	16.1	Not reached	2-yr OS 73.3%	17.1	[33,40,41]
Pembrolizumab/KEYNOTE-629	II	Locally advanced; recurrent or metastatic	159	75.5; 72	50; 35.2	16.7; 10.5	Not reached	12-mo OS 61%	11.9	[43]
Pembrolizumab/CARSKIN	II	First-line unresectable	57	79	42	7	Not reached	60% disease control at week 15	7	[42]
Pacmilimab/PROCLAIM-CX-072	I	Advanced solid tumors (cSCC subset)	14	Not reported	36	7	Not reported	Survival outcomes not formally reported	9 (overall cohort)	[44]

PD-1, programmed cell death protein 1; PD-L1, programmed death-ligand 1; cSCC, cutaneous squamous cell carcinoma; ORR, objective response rate; CR, complete response; DOR, duration of response; TRAEs, treatment-related adverse events; yr, year; mo, month; OS, overall survival; I, phase I clinical trial; II, phase II clinical trial; I/II, phase I/II clinical trial.

**Table 2:** Real-world outcomes of PD-1 inhibitors in advanced cSCC.

Region	Study Design/Agent(s)	*n*	Median Age (Years)	ECOG ≥ 2 (%)	ORR (%)	Survival Outcomes	Grade ≥ 3 AEs (%)	Key Notes	Reference
Europe (Germany)	Multicenter retrospective; pembrolizumab, nivolumab, or cemiplimab	46	76	NR	58.7% (CR 15.2%)	1-yr PFS 58.8%	13%	<10% discontinued due to toxicity	[45]
Europe (France)	Multicenter retrospective; cemiplimab	245	77	27	50.4% (CR 21%)	1-yr OS 63.1%	9%	1 fatal TEN	[46]
Europe (France, older)	Single-center retrospective; cemiplimab	22	83	27	32% (CR 9%)	NR	40.9%	High discontinuation (41%) due to toxicity	[47]
Europe (Italy)	Single-center retrospective (frail cohort); cemiplimab	30	81	20	76.7% (CR 30%)	Median PFS 16 mo; OS 18 mo	10%	83% frail, responses observed in immunocompromised	[48]
United States	Single institution, retrospective; cemiplimab, nivolumab, or pembrolizumab	26	64.5	15.4	42.3% (CR 23.1%)	Median PFS 5.4 mo	19.2%	Higher TMB and head/neck primary associated with response	[49]
United States	Multicenter retrospective incl. hematological malignancy; anti-PD-1 (cSCC subset)	15 (cSCC subset)	NR	NR	26.7% (CR 6.7%)	Median PFS 4.0 mo; OS 14.9 mo	NR	Shorter benefit in hematologic malignancies	[50]
Latin America	Multicenter retrospective; nivolumab	24	74	0	58.3%	Median PFS 12.7 mo; OS 20.7 mo	25%	No CRs; feasibility demonstrated	[51]
Australia	Multicenter retrospective; cemiplimab or pembrolizumab	286	75.2	21	60% (CR 27%)	1-yr OS 78%	NR	31% immunocompromised	[52]
Australia	Single-center retrospective; cemiplimab or pembrolizumab	53	81.8 (range 70.1–96.8)	34	57% (CR 33%)	1-yr OS 63%; PFS 41%	3.8%	34% immunocompromised; worse OS and PFS in poorer ECOG PS	[53]
Asia (Japan)	Single-center retrospective; pembrolizumab or nivolumab	14	64.5	7	57.1% (CR 14.3%)	1-yr OS 73.1%; 1-yr PFS 56.2%	7.1%	Small cohort; findings require validation in larger studies	[54]

ORR, objective response rate; AEs, adverse event; CR, complete response; OS, overall survival; PFS, progression-free survival; TEN, toxic epidermal necrolysis; TMB, tumor mutational burden; ECOG, Eastern Cooperative Oncology Group; PS, performance status; PD-1, programmed cell death protein 1; cSCC, cutaneous squamous cell carcinoma; mo, month; yr, year; NR, not reported. Variables and safety outcomes are presented as reported in the original publications and generally refer to grade ≥3 adverse events, including treatment-related or immune-related events when specified.

## Data Availability

No new data were created or analyzed in this study. Data sharing is not applicable to this article.

## Abbreviations

The following abbreviations are used in this manuscript:

ICIs	immune checkpoint inhibitors
PD-1	programmed cell death protein 1
MDSCs	myeloid-derived suppressor cells
TME	tumor microenvironment
IL	interleukin
TGF	transforming growth factor
Tregs	regulatory T cells
APCs	antigen-presenting cells
UV	ultraviolet
EGFR	epidermal growth factor receptor
ORR	objective response rate
DOR	duration of response
OS	overall survival
ECOG	Eastern Cooperative Oncology Group
PS	performance status
irAEs	immune-related adverse events
HBV	hepatitis B virus
PD-L1	programmed death-ligand 1
G8	Geriatric-8
TMB	tumor mutational burden
